# Salivary Antioxidant and Peroxidase Activity as a Marker of Steroid Hormone Receptor Expression in Breast Cancer

**DOI:** 10.3390/ijms27020587

**Published:** 2026-01-06

**Authors:** Elena A. Sarf, Lyudmila V. Bel’skaya

**Affiliations:** Biochemistry Research Laboratory, Omsk State Pedagogical University, 644099 Omsk, Russia; sarf_ea@omgpu.ru

**Keywords:** saliva, breast cancer, phenotypes, estrogen receptors, antioxidant activity, peroxidase, lipid peroxides, oxidative stress

## Abstract

The growth and development of breast cancer are accompanied by an increase in oxidative stress. A close relationship is known to exist between the biological activity of several antioxidant enzymes and the regulation of estrogen-mediated signaling in breast cancer. The aim of this study was to study the activity of salivary antioxidant enzymes and the level of lipid peroxidation products in breast cancer before and after surgical treatment. The study included 115 patients with breast cancer (58.7 ± 10.9 years) and 60 healthy volunteers (51.8 ± 12.1 years). Saliva samples were obtained again from 53 patients 4 weeks after surgery. The content of lipid peroxidation products, catalase activity, total antioxidant activity (AOA) and total peroxidase activity (TPA) in saliva were analyzed before and after breast cancer surgery. An increase in lipid peroxidation products in saliva was observed with positive estrogen receptor expression. For the first time, it was shown that in patients with breast cancer, the levels of salivary TPA and AOA increased, which is likely due to the important role of the salivary glands in antioxidant protection. It can be speculated that the effectiveness of antioxidant defense was associated with estrogen and progesterone receptor expression and was reduced in prognostically unfavorable breast cancer phenotypes (non-luminal and triple-negative breast cancer).

## 1. Introduction

One of the most common mechanisms of breast cancer carcinogenesis is oxidative stress due to increased free radical formation and decreased antioxidant defense mechanisms [1]. In the body, there is an oxidative balance between the rate of free radical formation and removal, which performs a regulatory function and is of particular importance in carcinogenesis [2]. Oxidative stress metabolites, in particular reactive oxygen species (ROS), are synthesized by various metabolic pathways, including aerobic respiration in mitochondria [3], and act as procarcinogens, damaging cellular components such as lipids, proteins, and DNA [4,5]. Antioxidant enzymes, by controlling the concentration of free radicals in the cell, which regulate the rate of proliferative processes in the tumor, can influence the course and outcome of the disease [6]. A close relationship is known between the biological activity of a number of antioxidant enzymes and the regulation of estrogen-mediated signaling in breast cancer [7]. Prooxidant processes in mammary gland tissue are mainly associated with lipid peroxidation, since the mammary gland is abundantly surrounded by adipose tissue [8,9]. Specifically, an increase in oxidative stress markers (malondialdehyde and nitric oxide) compared to controls, along with a decrease in superoxide dismutase activity and reduced glutathione levels, was demonstrated in tissues obtained after surgical removal of breast tumors [10]. Elevated levels of lipid peroxidation markers were observed in the plasma or serum of patients with breast cancer [11,12]. Disease progression or response to treatment largely depends on the individual patient’s ability to neutralize lipid peroxidation products or ROS (e.g., hydroxyl radical) [13]. The role of oxidative stress markers in breast cancer has been described in detail in a number of reviews [14,15,16].

In recent years, saliva has become a popular diagnostic material for research and clinical trials [17,18,19]. Due to its availability, ease of collection, and the possibility of repeated non-invasive sampling, it is ideal for screening, diagnosis, or monitoring of many diseases [20]. Despite the fact that the use of saliva as a biological fluid has a number of limitations related to the composition of saliva, collection methods, storage rules, and interpretation of test results, saliva is actively used for scientific research [21]. In the oral cavity, ROS are produced in the epithelium and directly in saliva and also regulate the oral microbiota [22]. As in plasma and tissues, free radicals and ROS in saliva play an important role in redox signaling and are necessary for physiological functions [23]. The antioxidant system of saliva includes enzymes (peroxidase, catalase, superoxide dismutase, etc.) and molecular antioxidants (ascorbic and uric acids, vitamin E, glutathione, etc.) [24]. Antioxidant enzymes act as scavengers, namely superoxide dismutase (which dismutates the superoxide radical to hydrogen peroxide), catalase, and glutathione peroxidase (which convert hydrogen peroxide to water) [25]. Glutathione peroxidase, together with glutathione-S-transferase, is involved in the detoxification of fatty acid hydroperoxides [26]. Although changes in salivary antioxidant enzyme activity in oncological pathologies have been studied in detail, the relationship between the lipid peroxidation system and salivary antioxidant defense before and after breast cancer surgery has not yet been examined.

A hypothesis has been formulated that changes in lipid peroxidation and antioxidant defense parameters in saliva are closely linked to the molecular biological characteristics of breast cancer, and those specific antioxidant enzymes in saliva may increase their activity in compensation due to the involvement of the salivary glands in the oral antioxidant defense system. The relationship between saliva parameters and breast cancer can be observed through their changes after surgical removal of the tumor.

The aim of this study was to evaluate the activity of salivary antioxidant enzymes and the level of lipid peroxidation products in breast cancer before and after surgery.

## 2. Results

**Figure 1:**
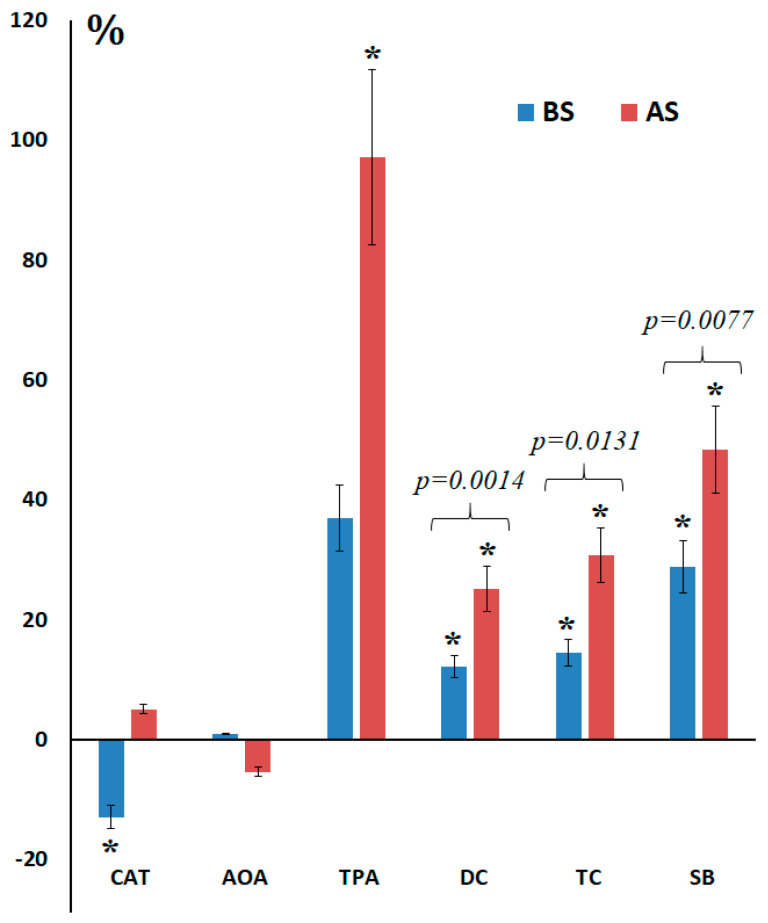
Comparison of salivary biochemical parameters before and after breast cancer surgery. BS—before surgery, AS—after surgery, CAT—catalase, AOA—antioxidant activity, TPA—total peroxidase activity, DC—diene conjugates, TC—triene conjugates, SB—Schiff bases. *—differences from healthy controls are statistically significant; *p*-values are given for comparison of groups before and after surgery. Here and further in Figure 1, Figure 2, Figure 3, Figure 4 and Figure 5, the values on the Y-axis were calculated as the value of the indicator for the main group minus the value of the corresponding indicator in the control group, divided by the value in the control group, and expressed as %. The error bars in Figure 1, Figure 2, Figure 3, Figure 4 and Figure 5 represent the IQR (25–75%).

In breast cancer patients, catalase activity in saliva decreased (−36.7%, *p* = 0.0017) while AOA increased (+37.0%) compared to the healthy control (Table 1, Figure 1).

The content of lipid peroxidation products increased: DC (+12.3%, *p* = 0.0156), TC (+14.6%, *p* = 0.0135) and SB (+28.8%, *p* = 0.0047). After surgery, catalase activity increased (+5.1%), AOA decreased (−5.3%), but AOA continued to increase (+97.2%, *p* = 0.0015) compared to the control group. A proportional increase in the content of lipid peroxidation products in saliva was also shown after breast cancer surgery: DC (+25.2%, *p* = 0.0004), TC (+30.8%, *p* = 0.0003) and SB (+48.4%, *p* = 0.0005) (Figure 1). Differences in the content of lipid peroxidation products before and after surgery were statistically significant (Figure 1).

**Figure 2:**
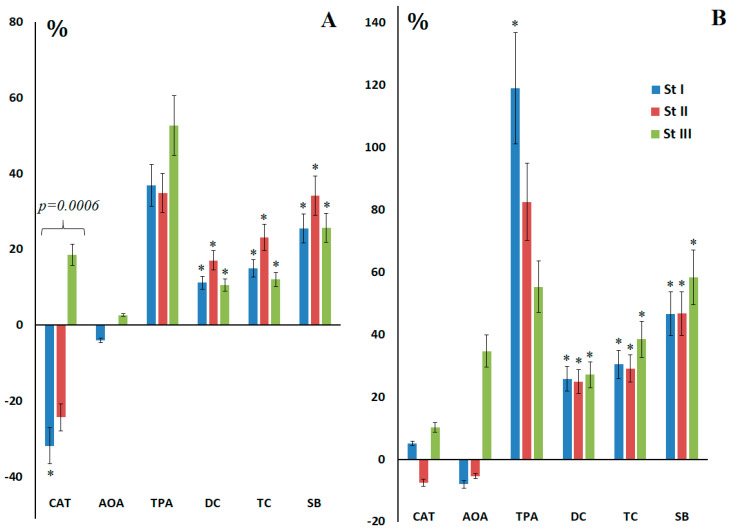
Relative activity of catalase, AOA, peroxidase, and the content of lipid peroxidation products in saliva compared with healthy controls before (**A**) and after (**B**) surgery depending on the stage of breast cancer. *—differences from healthy controls are statistically significant; *p*-value is given for comparison of stages I and III breast cancer.

The decrease in catalase and AOA before surgery was inversely proportional to the breast cancer stage (Figure 2A). Thus, the minimum catalase activity (−31.8%, *p* = 0.0484) and AOA (−4.0%) were observed for stage I, while at stage III an increase in both indicators was observed (+18.6% and +2.7%, respectively). Peroxidase activity was higher at all stages than in the healthy control (Figure 2A). After breast cancer surgery, catalase, AOA, and TPA activities increased, with catalase activity (+10.2%) and AOA (+34.7%) being higher at stage III, while the maximum increase in TPA was observed at stage I breast cancer (+118.9%, *p* = 0.0012) (Figure 2B). For lipid peroxidation products before surgery, an increase was noted in stage II: DC (+17.1%, *p* = 0.0328), TC (+23.1%, *p* = 0.0028) and SB (+34.2%, *p* = 0.0095) (Figure 2A). After surgery, the content of lipid peroxidation products was higher in stage III breast cancer: DC (+27.1%, *p* = 0.0067), TC (+38.5%, *p* = 0.0059) and SB (+58.4%, *p* = 0.0090) (Figure 2B).

**Figure 3:**
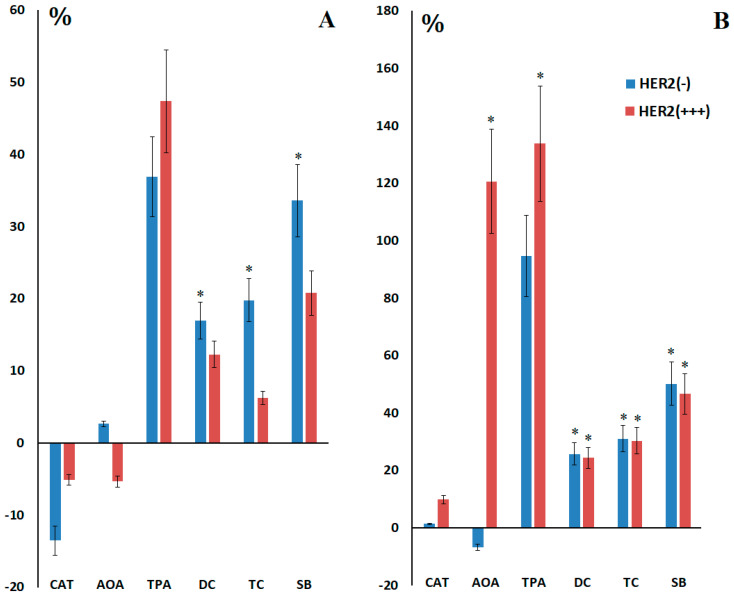
Relative activity of catalase, AOA, peroxidase, and the content of lipid peroxidation products in saliva compared to the control group before (**A**) and after (**B**) surgery depending on HER2 expression. *—differences with healthy controls are statistically significant.

Before breast cancer surgery, there were pronounced differences in the content of lipid peroxidation products in saliva between the subgroups with different expression of HER2 receptors (Figure 3A). Thus, with positive HER2 expression, the level of lipid peroxidation products in saliva is lower and does not show statistically significant differences from the healthy control (Figure 3A). After surgery, the content of lipid peroxidation products levels out, which means a more intense increase in DC (+24.4%, *p* = 0.0272), TC (+30.4%, *p* = 0.0271) and SB (+46.8%, *p* = 0.0202) in the subgroup with positive HER2 expression (Figure 3B). Statistically significant increase in saliva AOA after surgery with positive HER2 expression (+120.7%, *p* = 0.0345) (Figure 3).

**Figure 4:**
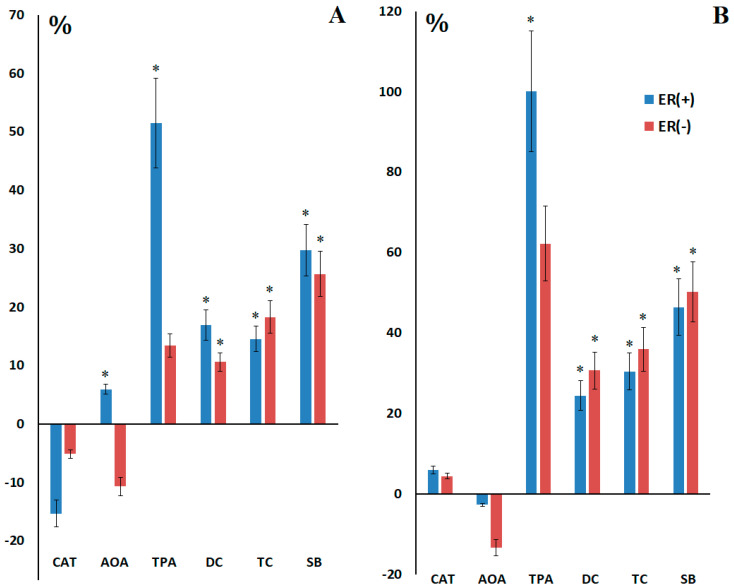
Relative activity of catalase, AOA, peroxidase, and the content of lipid peroxidation products in saliva compared to the control group before (**A**) and after (**B**) surgery depending on ER expression. *—differences with healthy controls are statistically significant.

Before surgery, statistically significantly higher AOA (+6.0%, *p* = 0.0263) and TPA (+51.5%, *p* = 0.0446) were observed in the subgroup with positive expression of estrogen receptors (Figure 4A). After surgery, this subgroup showed an increase in the content of lipid peroxidation products, which was statistically significant compared to the preoperative values: DC (+7.6%, *p* = 0.0167), TC (+15.9%, *p* = 0.0338) and SB (+16.7%, *p* = 0.0181) (Figure 4B). It should be noted that lower values of antioxidant enzyme activity are observed in the subgroups with higher lipid peroxidation levels after surgery (Figure 4B).

**Figure 5:**
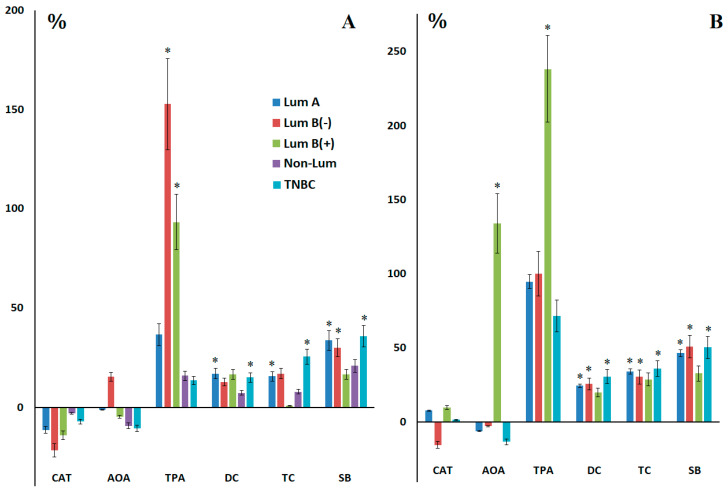
Relative activity of catalase, AOA, peroxidase, and the content of lipid peroxidation products in saliva compared to the control group before (**A**) and after (**B**) surgery depending on breast cancer phenotypes. *—differences with healthy controls are statistically significant.

A decrease in salivary catalase activity was observed for all breast cancer phenotypes before surgery (Figure 5A). The maximum decrease was noted for estrogen-hormone positive subtypes (−11.4%, −21.6% and −14.1% for Lum A, Lum B(−) and Lum B(+), respectively). Salivary AOA also decreased, but in this case the maximum decrease was observed in estrogen-hormone negative subtypes (−9.3% and −10.7% for Non-Lum and TNBC, respectively) (Figure 5A). The increase in salivary AOA was more pronounced in those subgroups where catalase activity decreased more significantly (Figure 5A). The maximum increase in the TPA was observed in the subgroups of Lum B(−) (+152.8%, *p* = 0.0016) and Lum B(+) (+93.3%, *p* = 0.0128) breast cancer and was statistically significant (Figure 5A).

When analyzing the patterns of changes in the level of lipid peroxidation products in saliva for the Lum A and Lum B(−) subtypes, no differences were shown (Figure 5A). For Lum B(+), with a high content of primary lipid peroxidation products–diene conjugates (+16.4%), the content of triene conjugates sharply decreased (+0.8%), and then the content of the most toxic Schiff bases increased (+16.5%). For Non-Lum A and TNBC, a uniform increase in the level of lipid peroxidation products in saliva from primary to Schiff bases was shown (Figure 5A). The level of lipid peroxidation products in saliva in TNBC was the highest (+15.0%, *p* = 0.0194; +25.4%, *p* = 0.0056 and +35.9%, *p* = 0.0097 for DC, TC and SB, respectively). Postoperatively, increases in catalase, AOA, and OPA activity were observed only in Lum B (+) breast cancer (Figure 5B). This subtype also had the lowest levels of lipid peroxidation products. Patients with Non-Lum breast cancer were referred for neoadjuvant chemotherapy, so postoperative data were not available for this subgroup.

## 3. Discussion

The role of lipid peroxidation in breast cancer has not been fully studied. A number of studies have shown elevated levels of various lipid peroxidation markers (malondialdehyde, 8-F2-isoprostanes, or 4-hydroxynonenal) in plasma, serum, urine, and tissues in breast cancer [27,28,29,30]. The content of lipid peroxidation products in breast tissue was significantly higher, which was accompanied by a significant increase in the amount of both enzymatic and non-enzymatic antioxidants compared to healthy controls [31]. Thus, patients with breast cancer have more pronounced lipid peroxidation, and the antioxidant system is activated as an adaptive mechanism.

The significant role of lipids in the diagnosis and prognosis of breast cancer is known [32,33,34]. In particular, the first signature based on lipid metabolism has been identified that can be used to predict and guide immunotherapy or chemotherapy in ER+ breast cancer [32]. Thus, arachidonate-15-lipoxygenase expression was positively correlated with larger tumor size, more advanced tumor stage, and vascular invasion [32]. A multidimensional signature of 20 plasma lipid biomarkers has been developed to distinguish healthy controls from breast cancer patients based on high-resolution liquid chromatography tandem mass spectrometry [33]. Lipids have been shown to be associated with breast cancer risk, and lipid-lowering medications may be effective in breast cancer prevention [34]. However, when we consider lipids and lipid peroxidation processes in the oral cavity, it must be kept in mind that only about 2% of lipids enter saliva from blood serum (cholesterol and free fatty acids) [35,36]. It is known that lipids enter the oral cavity mainly with the secretions of the parotid and submandibular salivary glands [37]. The likelihood of specific tumor products getting into saliva is extremely low. Therefore, the lipid composition of saliva reflects the body’s systemic inflammatory/immune response to a specific cancer subtype. The sources of a number of lipids in saliva are also the membranes of secretory vesicles and fragments of bacterial cell membranes. Lipids are one of the main components of cell membranes; therefore, changes in the lipid composition of saliva may reflect changes in the composition of the cell membranes of the salivary glands [38].

We have shown an increase in the level of lipid peroxidation products in saliva in all cases, with the most intense increase observed after breast cancer surgery. In this case, activation of lipid peroxidation processes can be considered as a leading pathogenetic factor in the destabilization of cell membranes and disruption of intercellular interactions. Moreover, the content of lipid peroxidation products in saliva before treatment is closely associated with the HER2 status of the tumor. Higher lipid peroxide levels are characteristic of HER2-negative breast tumors. After treatment, differences in lipid peroxidation levels between subgroups leveled out, but overall, lipid peroxide concentrations in saliva remained higher.

Lipid peroxidation processes are known to be activated after surgery, especially in the early postoperative period [39]. This is due to surgical stress, which leads to excessive formation of lipid peroxidation products, which have a toxic effect on membrane and intracellular structures. This postoperative surge is a temporary physiological response to surgical trauma and the subsequent healing process. However, there are known cases where persistent systemic oxidative imbalance persisted even after tumor removal. In particular, normalization of metabolic status after radical surgery in patients with edematous-infiltrative breast cancer did not occur in some cases [40]. A four-week observation period represents the late postoperative period, when the effects of surgical trauma should have abated. However, we did not observe a return of lipid peroxidation levels to preoperative values, indicating both the need for an extended observation period and additional correction of the oxidative imbalance.

An increase in lipid peroxidation products in saliva has been noted with positive expression of estrogen receptors, accompanied by a statistically significant increase in AOA and TPA. Estrogen receptors are known to be present in biopsies of normal tissues taken from the cheek, parotid gland, submandibular gland, and minor labial salivary glands. Specific nuclear receptors are localized in the basal layer of gingival epithelium, periodontal ligament fibroblasts, endothelial cells of periodontal vessels and oral mucosa, and alveolar and jaw bone cells [41,42]. This suggests that estrogen plays a biological role in homeostasis and normal functioning of the salivary glands and oral mucosa [43]. Estrogen receptor beta is the predominant estrogen receptor subtype in the salivary glands and oral mucosa [44].

It has been suggested that the role of oxidative stress in estrogen-receptor-positive breast cancer may differ from that in other tumor types [45]. Several studies have demonstrated in vitro that mitochondrial ROS can be induced by physiological concentrations of estrogen [46,47]. Because oxidative metabolism of estrogen and subsequent ROS generation are key estrogen-associated carcinogenic mechanisms [48,49], ROS scavenging systems are expected to play a particularly important role in estrogen-receptor-positive malignancies. The oxidative effect of estrogen is associated with the production of ROS from unstable compounds such as semiquinones, formed through tissue-specific conversion of estrogen to catechol estrogen metabolites [46]. Estrogen-mediated oxidative stress may result from altered antioxidant enzyme status [50], in particular, estrogens affect the activity of glutathione transferase, glutathione peroxidase, superoxide dismutase, and catalase [51]. Estrogen and its metabolites can have the same damaging effects as ROS, alkylating or damaging the integrity of DNA and proteins [52], and also binding to estrogen receptors and activating elements of the estrogen response, which leads to increased ROS levels [53]. Under these conditions, even increased activity of cellular antioxidant enzymes does not protect macromolecules from the effects of oxidative stress in breast cancer. These results indicate an increased need for antioxidants in breast cancer [54].

The antioxidant properties of saliva are mediated by a combination of various molecular mechanisms [55,56,57]. For example, catalase plays an important role because it mediates NADPH-dependent dismutation of hydrogen peroxide into water and oxygen [58]. Hydrogen peroxide can be produced and released into saliva by oral microbiota [59]. Hydrogen peroxide can also penetrate cell membranes and serve as a second messenger in many cellular processes [60]. Traditionally, catalase activity measured in saliva is attributed primarily to bacteria. More recent proteomic studies have shown that catalase activity in saliva is of both human and bacterial origin [61].

We have shown a decrease in salivary catalase activity in all cases before breast cancer surgery, which may indicate a decrease in its production by the oral microbiota and a decrease in its intake from the plasma. This leads to the need to enhance other links in the antioxidant defense of saliva, in particular peroxidase. Two peroxidases are present in saliva: lactoperoxidase, produced by the parotid and submandibular glands, and myeloperoxidase, contained in polymorphonuclear neutrophils [62]. These enzymes not only reduce hydrogen peroxide, but also have an antimicrobial effect due to the oxidation of thiocyanate ion [63]. The resulting hypothiocyanate ion limits the proliferation of bacteria in the oral cavity by oxidizing thiol residues in the main microbial proteins [64], and can also inactivate proteins involved in the detoxification of human saliva [65]. Thus, antioxidant systems play an important role in maintaining the redox balance of saliva.

ROS are known to be essential for immune responses, but their excessive production triggers and maintains pro-inflammatory processes, particularly through the redox-sensitive transcription factors nuclear factor (erythroid 2)-like 2 (Nrf2) and nuclear factor κB (NF-κB) [66]. Inflammation further enhances oxidative stress by increasing ROS production and myeloperoxidase release, which leads to oxidative damage to lipids, nucleic acids, and proteins and promotes tissue damage [66]. Salivary myeloperoxidase levels are dependent on two main factors: the natural migration of neutrophils into saliva and the inflammatory response of the mucous membranes in oral diseases. It can be speculated that salivary myeloperoxidase levels may reflect systemic changes in the body, just as oral health affects overall health [67].

Lactoperoxidase is involved in the one-electron oxidation of 17β-estradiol to the reactive phenoxyl radical [14]. Lactoperoxidase plays a role in breast carcinogenesis through the activation of carcinogenic aromatic amines such as benzidine, 2-aminofluorene, and others, resulting in the formation of metabolites that are highly reactive and covalently bind to DNA [68].

An increase in the activity of lactoperoxidase, as the only antioxidant synthesized in the salivary glands, reflects the effectiveness of the salivary glands in preventing oxidative stress. An increase in peroxidase activity in the saliva of patients with breast cancer indicates an increase in enzymatic antioxidant defense, which protects the salivary glands and the entire oral cavity from oxidative damage. An increase in catalase activity is associated with a positive status of HER2 receptor expression and a negative status of hormone receptor expression. An increase in AOA and TPA is associated with a positive expression of estrogen receptors. This pattern persists after surgical removal of the tumor. It is known that breast cancer subtypes are characterized by different ROS production and susceptibility to antioxidant treatment [69]. We have shown that salivary catalase activity decreases more significantly in luminal breast cancer subtypes (Lum A, Lum B(−) and Lum B(+)), while AOA decreases to a greater extent in HER2-positive (Lum B(+) and Non-Lum) and TNBC. It is known that estrogens and their metabolites are capable of altering the redox balance through increased generation of superoxide radicals with their signaling and damaging effects [70,71], which may cause a more noticeable depletion of antioxidant protection in patients with estrogen-positive (liminal) breast cancer [72].

Thus, saliva is suitable for assessing oxidative stress levels, enabling non-invasive diagnostics of both systemic disorders in the body, including those caused by breast cancer, and localized disorders in the oral cavity [73]. The oral mucosa reflects metabolic disorders and pathologies of individual organs and systems [74]. Monitoring the activity of salivary antioxidant enzymes can identify subgroups of patients with impaired antioxidant defense systems and identify risk groups for developing oral complications during breast cancer treatment [75]. Specifically, correcting decreased oral antioxidant defenses may involve the use of medications, physical therapy, hygiene products, and, in some cases, dietary modifications [76,77,78]. The use of antioxidants is considered a promising approach for correcting metabolic disorders that develop as a result of chemotherapy for breast cancer [79,80].

Study limitations include an incomplete list of salivary antioxidant enzymes measured (glutathione peroxidase, superoxide dismutase, and others were not included), as well as the lack of analysis of the non-enzymatic components of salivary antioxidant defense (albumin, uric acid, and others). The observation period after breast cancer surgery was only 4 weeks, and subsequent measurements were not possible due to the initiation of adjuvant chemotherapy. Volunteers did not receive any medications for the treatment of breast cancer, but it was not possible to completely exclude the use of drugs for chronic comorbidities, which can also be considered a limitation of the study.

The small sample size and the lack of samples collected at multiple centers are among the study’s limitations. The small sample size is due to the fact that some patients received prior chemotherapy after the diagnostic checkup or had contraindications for surgery, resulting in loss to follow-up and the failure to obtain a repeat saliva sample after surgery. A larger cohort is planned for continued research.

## 4. Materials and Methods

### 4.1. Study Design

This study involved 115 patients with breast cancer (58.7 ± 10.9 years) and 60 healthy volunteers (51.8 ± 12.1 years). Volunteers were recruited at the Omsk Clinical Oncology Dispensary: breast cancer patients were recruited in the emergency department, and healthy controls were recruited in the blood transfusion department. Healthy volunteers were active blood donors and underwent a preliminary examination by a physician to rule out contraindications. The criterion for inclusion in the healthy control group was the absence of breast abnormalities during routine mammography and/or ultrasound.

The breast cancer group included patients strictly prior to treatment with no signs of active infection or inflammatory processes in the oral cavity. Histological verification of the diagnosis was considered a prerequisite. Tumor tissue expression was determined for estrogen (ER) and progesterone (PR) receptors [81], human epidermal growth factor 2 (HER2) [82], and the Ki-67 proliferative activity index [83].

A detailed description of the group of patients with breast cancer is given in Table 1. No differences in age were found between subgroups with different stages and phenotypes of breast cancer. Patients with stage IV did not undergo surgery; therefore, these patients were excluded from further analysis. For patients with non-luminal breast cancer, surgery was performed only after neoadjuvant chemotherapy; for this subgroup, saliva samples were obtained before the first course of treatment (Table 2).

Saliva collection was performed strictly before the start of anticancer drug therapy: twice during hospitalization for surgery (Before Surgery) and before the start of the first course of adjuvant chemotherapy (After Surgery).

### 4.2. Saliva Biochemical Analysis

Saliva samples were obtained as previously described [84,85]. Saliva samples were collected between 8 and 10 a.m. (the time of peak salivary secretion) after an overnight fast, after rinsing the mouth with water without stimulation. A tooth brushing was permitted no later than one hour before sample collection. All volunteers were instructed to abstain from alcohol consumption for two days prior to saliva collection and from smoking and taking medications from the moment of awakening until saliva collection. Samples were collected by spitting into sterile polypropylene tubes and centrifuged to reduce turbidity and remove cellular elements at 10,000× *g* for 10 min (CLb-16, Moscow, Russia). Saliva analysis was performed immediately after collection without storage or freezing.

All saliva samples were analyzed for catalase activity (nkat/mL), total antioxidant activity (AOA, mmol/L), total peroxidase activity (TPA, c.u.), and the content of lipid peroxidation products—diene conjugates (DC, c.u.), triene conjugates (TC, c.u.), and Schiff bases (SB, c.u.).

Determination of AOA was carried out using ready-made commercial Vector-Best kits (cat. No. B-7501, Novosibirsk, Russia) using the semi-automatic biochemical analyzer StatFax 3300 (Awareness Technology, Palm City, FL, USA). Catalase activity was determined using a commercial Servicebio kit (cat. No. G4307-48T, Wuhan Servicebio Technology, Wuhan, China). TPA was determined using a commercial Ecotech kit (Moscow, Russia). The levels of lipid peroxidation products were determined spectrophotometrically using the Volchegorsky method [86].

### 4.3. Statistical Analysis

Statistical analysis of the obtained data was performed using Statistica 13.3 EN software (StatSoft, Tulsa, OK, USA) using a nonparametric method. The distribution and homogeneity of variances in the groups were preliminarily checked using the Shapiro–Wilk and Bartlett tests. The sample was described using the median (Me) and interquartile range as the 25th and 75th percentiles [LQ; UQ]. Differences were considered statistically significant at *p* < 0.05.

## 5. Conclusions

Increased levels of lipid peroxidation products in saliva are associated with negative HER2 expression status and positive estrogen receptor expression status. In the same subgroups, a decrease in salivary catalase activity was noted, while an increase in AOA and TPA was associated only with positive expression of estrogen receptors. This pattern persists after surgical removal of the tumor. Thus, for the first time it has been shown that peroxidase and antioxidant activity in the saliva of breast cancer patients increases, demonstrating the important role of the salivary glands in antioxidant defense. We hypothesize that the effectiveness of antioxidant defense is associated with the expression of estrogen and progesterone receptors and is reduced in prognostically unfavorable breast cancer subtypes (Non-Lum and TNBC).

## Figures and Tables

**Table 1:** Biochemical parameters of saliva before and after breast cancer surgery and in healthy controls.

Indicators	Breast Cancer		Healthy Controls (3)
	Before Surgery (1)	After Surgery (2)	
Catalase, nkat/L	2.90 [1.96; 3.99]	3.50 [2.44; 3.98]	3.33 [2.23; 4.31]
	*p1–3 = 0.0017*	-	*p1–3 = 0.0017*
AOA, mmol/L	2.14 [1.74; 3.05]	2.03 [1.83; 2.91]	2.14 [1.75; 2.50]
TPA, c.u.	1.48 [0.92; 2.86]	2.13 [1.08; 3.63]	1.08 [0.59; 2.14]
	-	*p2–3 = 0.0015*	*p2–3 = 0.0015*
DC, c.u.	3.01 [2.72; 3.36]	3.35 [3.05; 3.50]	2.68 [2.59; 2.80]
	*p1–3 = 0.0156*	*p2–3 = 0.0004*	*p1–3 = 0.0156*
	*p1–2 = 0.0014*	*p1–2 = 0.0014*	*p2–3 = 0.0004*
TC, c.u.	1.66 [1.44; 1.98]	1.89 [1.66; 2.05]	1.45 [1.24; 1.52]
	*p1–3 = 0.0135*	*p2–3 = 0.0003*	*p1–3 = 0.0135*
	*p1–2 = 0.0131*	*p1–2 = 0.0131*	*p2–3 = 0.0003*
SB, c.u.	0.932 [0.835; 1.089]	1.074 [0.940; 1.248]	0.724 [0.665; 0.898]
	*p1–3 = 0.0047*	*p2–3 = 0.0005*	*p1–3 = 0.0047*
	*p1–2 = 0.0077*	*p1–2 = 0.0077*	*p2–3 = 0.0005*

Note. *p*-values are given for comparison of groups designated by numbers: 1—breast cancer before surgery, 2—breast cancer after surgery, 3—healthy control.

**Table 2:** Description of breast cancer subgroups.

Subgroups		Before Surgery, n (%)	After Surgery, n (%)
Age, years		58.7 ± 10.9	59.1 ± 10.2
*Stage*			
	IA + IB	40 (34.8%)	25 (47.2%)
	IIA + IIB	45 (39.1%)	19 (35.8%)
	IIIA + IIIB + IIIC	26 (22.6%)	9 (17.0%)
	IV	4 (3.5%)	-
*Breast Cancer Phenotype*			
	Luminal A = Lum A	40 (34.8%)	10 (18.9%)
	Luminal B HER2-negative = Lum B(−)	36 (31.3%)	25 (47.2%)
	Luminal B HER2-positive = Lum B(+)	11 (9.6%)	8 (15.0%)
	Non-luminal HER2-positive = Non-Lum	10 (8.7%)	-
	Triple negative breast cancer = TNBC	18 (15.6%)	10 (18.9%)

## Data Availability

The data presented in this study are available on request from the corresponding author (The raw data supporting the conclusions of this article will be made available by the authors on request).

## References

[1] Ramírez-Expósito M.J., Sánchez-López E., Cueto-Ureña C., Dueñas B., Carrera-González P., Navarro-Cecilia J., Mayas M.D., Arias de Saavedra J.M., Sánchez-Agesta R., Martínez-Martos J.M. (2014). Circulating oxidative stress parameters in pre- and post-menopausal healthy women and in women suffering from breast cancer treated or not with neoadjuvant chemotherapy. Exp. Gerontol..

[2] Jelic M.D., Mandic A.D., Maricic S.M., Srdjenovic B.U. (2021). Oxidative stress and its role in cancer. J. Cancer Res. Ther..

[3] Alpay M., Backman L.R., Cheng X., Dukel M., Kim W.J., Ai L., Brown K.D. (2015). Oxidative stress shapes breast cancer phenotype through chronic activation of ATM-dependent signaling. Breast Cancer Res. Treat..

[4] Markovsky A.V. (2023). Features of aminothiol metabolism and progression of breast cancer. Sib. J. Oncol..

[5] Forman H.J., Zhang H. (2021). Targeting oxidative stress in disease: Promise and limitations of antioxidant therapy. Nat. Rev. Drug Discov..

[6] Jomova K., Alomar S.Y., Valko R., Fresser L., Nepovimova E., Kuca K., Valko M. (2025). Interplay of oxidative stress and antioxidant mechanisms in cancer development and progression. Arch. Toxicol..

[7] O’Leary P.C., Terrile M., Bajor M., Gaj P., Hennessy B.T., Mills G.B., Zagozdzon A., O’Connor D.P., Brennan D.J., Connor K. (2014). Peroxiredoxin-1 protects estrogen receptor α from oxidative stress-induced suppression and is a protein biomarker of favorable prognosis in breast cancer. Breast Cancer Res..

[8] Pan S.Y., Zhou J., Gibbons L., Morrison H., Wen S.W. (2011). Canadian Cancer Registries Epidemiology Research Group [CCRERG]. Antioxidants and breast cancer risk- a population-based case-control study in Canada. BMC Cancer.

[9] Mencalha A., Victorino V.J., Cecchini R., Panis C. (2014). Mapping oxidative changes in breast cancer: Understanding the basic to reach the clinics. Anticancer Res..

[10] Qebesy H.S., Zakhary M.M., Abd-Alaziz M.A., Abdel Ghany A.A., Maximus D.W. (2015). Tissue levels of oxidative stress markers and antioxidants in breast cancer patients in relation to tumor grade. AAMJ.

[11] Mazzuferi G., Bacchetti T., Islam M.O., Ferretti G. (2021). High density lipoproteins and oxidative stress in breast cancer. Lipids Health Dis..

[12] Ghafoor D.D. (2023). Correlation between oxidative stress markers and cytokines in different stages of breast cancer. Cytokine.

[13] Jablonska E., Gromadzinska J., Peplonska B., Fendler W., Reszka E., Krol M.B., Wieczorek E., Bukowska A., Gresner P., Galicki M. (2015). Lipid peroxidation and glutathione peroxidase activity relationship in breast cancer depends on functional polymorphism of GPX1. BMC Cancer.

[14] Nourazarian A.R., Kangari P., Salmaninejad A. (2014). Roles of oxidative stress in the development and progression of breast cancer. Asian Pac. J. Cancer Prev..

[15] Hecht F., Pessoa C.F., Gentile L.B., Rosenthal D., Carvalho D.P., Fortunato R.S. (2016). The role of oxidative stress on breast cancer development and therapy. Tumour Biol..

[16] Bel’skaya L.V., Dyachenko E.I. (2024). Oxidative Stress in Breast Cancer: A Biochemical Map of Reactive Oxygen Species Production. Curr. Issues Mol. Biol..

[17] Nonaka T., Wong D.T.W. (2023). Saliva diagnostics: Salivaomics, saliva exosomics, and saliva liquid biopsy. J. Am. Dent. Assoc..

[18] Surdu A., Foia L.G., Luchian I., Trifan D., Tatarciuc M.S., Scutariu M.M., Ciupilan C., Budala D.G. (2025). Saliva as a Diagnostic Tool for Systemic Diseases-A Narrative Review. Medicina.

[19] Carneiro M.C., da Silva N.D.G., Ventura T.M.O., da Silva Santos P.S., Buzalaf M.A.R., Freire M. (2026). Salivary Proteome Role in Infection and Immunity. Adv. Exp. Med. Biol..

[20] Li Y., Ou Y., Fan K., Liu G. (2024). Salivary diagnostics: Opportunities and challenges. Theranostics.

[21] Mortazavi H., Yousefi-Koma A.A., Yousefi-Koma H. (2024). Extensive comparison of salivary collection, transportation, preparation, and storage methods: A systematic review. BMC Oral Health.

[22] Schwartz M., Neiers F., Feron G., Canon F. (2021). The Relationship Between Salivary Redox, Diet, and Food Flavor Perception. Front. Nutr..

[23] Tóthová L., Kamodyová N., Červenka T., Celec P. (2015). Salivary markers of oxidative stress in oral diseases. Front. Cell. Infect. Microbiol..

[24] Żukowski P., Maciejczyk M., Waszkiel D. (2018). Sources of free radicals and oxidative stress in the oral cavity. Arch. Oral Biol..

[25] Shlapakova T.I., Kostin R.K., Tyagunova E.E. (2020). Reactive oxygen species: Their role in cellular processes and pathology. Bioorganic Chem..

[26] Cozza G., Rossetto M., Bosello-Travain V., Maiorino M., Roveri A., Toppo S., Zaccarin M., Zennaro L., Ursini F. (2017). Glutathione peroxidase 4-catalyzed reduction of lipid hydroperoxides in membranes: The polar head of membrane phospholipids binds the enzyme and addresses the fatty acid hydroperoxide group toward the redox center. Free Radic. Biol. Med..

[27] Gupta R.K., Patel A.K., Kumari R., Chugh S., Shrivastav C., Mehra S., Sharma A.N. (2012). Interactions between oxidative stress, lipid profile and antioxidants in breast cancer: A case control study. Asian Pac. J. Cancer Prev..

[28] Kedzierska M., Olas B., Wachowicz B., Jeziorski A., Piekarski J. (2010). The lipid peroxidation in breast cancer patients. Gen. Physiol. Biophys..

[29] Ray G., Batra S., Shukla N.K., Deo S., Raina V., Ashok S., Husain S.A. (2000). Lipid peroxidation, free radical production and antioxidant status in breast cancer. Breast Cancer Res. Treat..

[30] Rajneesh C.P., Manimaran A., Sasikala K.R., Adaikappan P. (2008). Lipid peroxidation and antioxidant status in patients with breast cancer. Singap. Med. J..

[31] Wang C., Yu J., Wang H., Zhang J., Wu N. (2014). Lipid peroxidation and altered anti-oxidant status in breast adenocarcinoma patients. Drug Res..

[32] Shen L., Huang H., Li J., Chen W., Yao Y., Hu J., Zhou J., Huang F., Ni C. (2023). Exploration of prognosis and immunometabolism landscapes in ER+ breast cancer based on a novel lipid metabolism-related signature. Front. Immunol..

[33] Li D., Heffernan K., Koch F.C., Peake D.A., Pascovici D., David M., Kehelpannala C., Mann G.B., Speakman D., Hurrell J. (2024). Discovery of Plasma Lipids as Potential Biomarkers Distinguishing Breast Cancer Patients from Healthy Controls. Int. J. Mol. Sci..

[34] Zhang Z., Zhang D. (2024). Circulating lipids, lipid-lowering drug targets, and breast cancer risk: Comprehensive evidence from Mendelian randomization and summary data-based Mendelian randomization. Cancer Causes Control.

[35] Caterino M., Fedele R., Carnovale V., Castaldo A., Gelzo M., Iacotucci P., Ruoppolo M., Castaldo G. (2023). Lipidomic alterations in human saliva from cystic fibrosis patients. Sci. Rep..

[36] Fineide F., Chen X., Bjellaas T., Vitelli V., Utheim T.P., Jensen J.L., Galtung H.K. (2021). Characterization of lipids in saliva, tears and minor salivary glands of Sjögren’s syndrome patients using an HPLC/MS-based approach. Int. J. Mol. Sci..

[37] Matczuk J., Żendzian-Piotrowska M., Maciejczyk M., Kurek K. (2017). Salivary lipids: A review. Adv. Clin. Exp. Med..

[38] Zięba S., Błachnio-Zabielska A., Maciejczyk M., Pogodzińska K., Szuta M., Lo Giudice G., Lo Giudice R., Zalewska A. (2024). Impact of Smoking on Salivary Lipid Profile and Oxidative Stress in Young Adults: A Comparative Analysis between Traditional Cigarettes, E-Cigarettes, and Heat-Not-Burn Products. Med. Sci. Monit..

[39] Smirnova O.V., Elmanova N.G. (2020). Interconditionality of the effects of types I and II of inflammation on the processes of lipid peroxidation and antioxidant protection in patients with obstructive jaundice of gallstone origin in the postoperative period. Immunologiya.

[40] Plokhov V.N., Chesnokova N.P. (2008). Patterns of paraneoplastic disorders in edematous-infiltrative form of breast cancer. Mod. Probl. Sci. Educ..

[41] Palanisamy S. (2025). The impact of estrogen on periodontal tissue integrity and inflammation-a mini review. Front. Dent. Med..

[42] Cornejo Ulloa P.E., Krom B.P., Schoonmade L.J., van der Veen M.H. (2024). Sex steroid hormones: An overlooked yet fundamental factor in oral homeostasis in humans. Front. Endocrinol..

[43] Maksimova M.Y. (2024). Burning mouth syndrome in menopausal women. Russ. J. Pain..

[44] Välimaa H., Savolainen S., Soukka T., Silvoniemi P., Mäkelä S., Kujari H., Gustafsson J.A., Laine M. (2004). Estrogen receptor-beta is the predominant estrogen receptor subtype in human oral epithelium and salivary glands. J. Endocrinol..

[45] Karihtala P., Kauppila S., Soini Y., Arja Jukkola V. (2011). Oxidative stress and counteracting mechanisms in hormone receptor positive, triple-negative and basal-like breast carcinomas. BMC Cancer.

[46] Sastre-Serra J., Valle A., Company M.M., Garau I., Oliver J., Roca P. (2010). Estrogen down-regulates uncoupling proteins and increases oxidative stress in breast cancer. Free Radic. Biol. Med..

[47] Felty Q., Xiong W.C., Sun D., Sarkar S., Singh K.P., Parkash J., Roy D. (2005). Estrogen-induced mitochondrial reactive oxygen species as signal-transducing messengers. Biochemistry.

[48] Okoh V., Deoraj A., Roy D. (2011). Estrogen-induced reactive oxygen species-mediated signalings contribute to breast cancer. Biochim. Biophys. Acta..

[49] Yager J.D., Davidson N.E. (2006). Estrogen carcinogenesis in breast cancer. N. Engl. J. Med..

[50] Pedram A., Razandi M., Wallace D.C., Levin E.R. (2006). Functional estrogen receptors in the mitochondria of breast cancer cells. Mol. Biol. Cell.

[51] Kushner P.J., Agard D.A., Greene G.L., Scanlan T.S., Shiau A.K., Uht R.M., Webb P. (2000). Estrogen receptor pathways to AP-1. J. Steroid Biochem. Mol. Biol..

[52] Cavalieri E.L., Rogan E.G. (2004). A unifying mechanism in the initiation of cancer and other diseases by catechol quinones. Ann. N. Y. Acad. Sci..

[53] Roy D., Liehr J.G. (1999). Estrogen, DNA damage and mutations. Mutat. Res..

[54] Zowczak-Drabarczyk M.M., Murawa D., Kaczmarek L., Połom K., Litwiniuk M. (2013). Total antioxidant status in plasma of breast cancer patients in relation to ERβ expression. Contemp. Oncol..

[55] Sivadasan P., Gupta M.K., Sathe G.J., Balakrishnan L., Palit P., Gowda H., Suresh A., Kuriakose M.A., Sirdeshmukh R. (2015). Human salivary proteome—A resource of potential biomarkers for oral cancer. J. Proteom..

[56] Grassl N., Kulak N.A., Pichler G., Geyer P.E., Jung J., Schubert S., Sinitcyn P., Cox J., Mann M. (2016). Ultra-deep and quantitative saliva proteome reveals dynamics of the oral microbiome. Genome Med..

[57] Pappa E., Vastardis H., Mermelekas G., Gerasimidi-Vazeou A., Zoidakis J., Vougas K. (2018). Saliva proteomics analysis offers insights on type 1 diabetes pathology in a pediatric population. Front. Physiol..

[58] Glorieux C., Calderon P.B. (2017). Catalase, a remarkable enzyme: Targeting the oldest antioxidant enzyme to find a new cancer treatment approach. Biol. Chem..

[59] Carlsson J. (1987). Salivary peroxidase: An important part of our defense against oxygen toxicity. J. Oral Pathol. Med..

[60] Stone J.R., Yang S. (2006). Hydrogen peroxide: A signaling messenger. Antioxid. Redox Signal..

[61] Denny P., Hagen F.K., Hardt M., Liao L., Yan W., Arellanno M., Bassilian S., Bedi G.S., Boontheung P., Cociorva D. (2008). The proteomes of human parotid and submandibular/sublingual gland salivas collected as the ductal secretions. J. Proteome Res..

[62] Ihalin R., Loimaranta V., Tenovuo J. (2006). Origin, structure, and biological activities of peroxidases in human saliva. Arch. Biochem. Biophys..

[63] Schaffer W.M., Bronnikova T.V. (2012). Peroxidase-ROS interactions. Nonlinear Dyn..

[64] Wang X., Ashby M.T. (2008). Reactive sulfur species: Kinetics and mechanism of the reaction of thiocarbamate-s-oxide with cysteine. Chem. Res. Toxicol..

[65] Fabrini R., Bocedi A., Camerini S., Fusetti M., Ottaviani F., Passali F.M., Topazio D., Iavarone F., Francia I., Castagnola M. (2014). Inactivation of human salivary glutathione transferase P1-1 by hypothiocyanite: A post-translational control system in search of a role. PLoS ONE.

[66] Muro P., Zhang L., Li S., Zhao Z., Jin T., Mao F., Mao Z. (2024). The emerging role of oxidative stress in inflammatory bowel disease. Front. Endocrinol..

[67] Nijakowski K., Jankowski J., Gruszczyński D., Surdacka A. (2023). Salivary Alterations of Myeloperoxidase in Patients with Systemic Diseases: A Systematic Review. Int. J. Mol. Sci..

[68] Sheikh I.A., Beg M.A., Yasir M. (2017). Molecular Interactions of Carcinogenic Aromatic Amines, 4-Aminobiphenyl and 4,4′-Diaminobiphenyl, with Lactoperoxidase—Insight to Breast Cancer. Anticancer Res..

[69] Sarmiento-Salinas F.L., Delgado-Magallón A., Montes-Alvarado J.B., Ramírez-Ramírez D., Flores-Alonso J.C., Cortés-Hernández P., Reyes-Leyva J., Herrera-Camacho I., Anaya-Ruiz M., Pelayo R. (2019). Breast Cancer Subtypes Present a Differential Production of Reactive Oxygen Species (ROS) and Susceptibility to Antioxidant Treatment. Front. Oncol..

[70] Cavalieri E.L., Rogan E.G. (2011). Unbalanced metabolism of endogenous estrogens in the etiology and prevention of human cancer. J. Steroid Biochem. Mol. Biol..

[71] Maiti S., Nazmeen A. (2019). Impaired redox regulation of estrogen metabolizing proteins is important determinant of human breast cancers. Cancer Cell Int..

[72] Arumalla K.K., Haince J.-F., Bux R.A., Huang G., Tappia P.S., Ramjiawan B., Ford W.R., Vaida M. (2024). Metabolomics-Based Machine Learning Models Accurately Predict Breast Cancer Estrogen Receptor Status. Int. J. Mol. Sci..

[73] Ghasemi S., Farokhpour F., Mortezagholi B., Movahed E., Ghaedi A., Gargari M.K., Khanzadeh M., Bazrgar A., Khanzadeh S. (2023). Systematic review and meta-analysis of oxidative stress and antioxidant markers in recurrent aphthous stomatitis. BMC Oral Health.

[74] Lavrovskaya Y.A., Romanenko I.G., Lavrovskaya O.M. (2019). Correction of antioxidant protection of the oral mucosa in chronic pancreatitis. Parodontologiya.

[75] Taichman L.S., Gomez G., Inglehart M.R. (2015). Oral Health-Related Complications of Breast Cancer Treatment: Assessing Dental Hygienists’ Knowledge and Professional Practice. J. Dent. Hyg..

[76] Durnovo E.A., Kontorshhikova K.N., Shakhova M.A., Soloveva A.G., Tarakanova V.A., Galkina E.S. (2021). Oxidation-reduction potential of tissues of the oral mucosal wound surface under the photodynamic action. Stomatologiia.

[77] Raza A., Karimyan N., Watters A., Emperumal C.P., Al-Eryani K., Enciso R. (2022). Efficacy of oral and topical antioxidants in the prevention and management of oral mucositis in head and neck cancer patients: A systematic review and meta-analyses. Support. Care Cancer.

[78] Azimi S., Mansouri Z., Bakhtiari S., Tennant M., Kruger E., Rajabibazl M., Daraei A. (2017). Does green tea consumption improve the salivary antioxidant status of smokers?. Arch. Oral. Biol..

[79] Gurer-Orhan H., Ince E., Konyar D., Saso L., Suzen S. (2018). The Role of Oxidative Stress Modulators in Breast Cancer. Curr. Med. Chem..

[80] Mdkhana B., Goel S., Saleh M.A., Siddiqui R., Khan N.A., Elmoselhi A.B. (2022). Role of oxidative stress in angiogenesis and the therapeutic potential of antioxidants in breast cancer. Eur. Rev. Med. Pharmacol. Sci..

[81] Ilić I.R., Stojanovi’c N.M., Radulović N.S., Živković V.V., Randjelović P.J., Petrović A.S., Božić M., Ilić R.S. (2019). The Quantitative ER Immunohistochemical Analysis in Breast Cancer: Detecting the 3 + 0, 4 + 0, and 5 + 0 Allred Score Cases. Medicina.

[82] Wolff A.C., Hammond M.E.H., Allison K.H., Harvey B.E., Mangu P.B., Bartlett J.M.S., Bilous M., Ellis I.O., Fitzgibbons P., Hanna W. (2018). Human Epidermal Growth Factor Receptor 2 Testing in Breast Cancer: American Society of Clinical Oncology/College of American Pathologists Clinical Practice Guideline Focused Update. J. Clin. Oncol..

[83] Stålhammar G., Robertson S., Wedlund L., Lippert M., Rantalainen M., Bergh J., Hartman J. (2018). Digital image analysis of Ki67 in hot spots is superior to both manual Ki67 and mitotic counts in breast cancer. Histopathology.

[84] Dyachenko E.I., Bel’skaya L.V. (2024). Salivary Metabolites in Breast Cancer and Fibroadenomas: Focus on Menopausal Status and BMI. Metabolites.

[85] Bel’skaya L.V., Sarf E.A., Solomatin D.V., Kosenok V.K. (2020). Diagnostic and Prognostic Value of Salivary Biochemical Markers in Oral Squamous Cell Carcinoma. Diagnostics.

[86] Bel’skaya L.V., Kosenok V.K., Massard G. (2016). Endogenous Intoxication and Saliva Lipid Peroxidation in Patients with Lung Cancer. Diagnostics.

